# Reparative macrophage transplantation for myocardial repair: a refinement of bone marrow mononuclear cell-based therapy

**DOI:** 10.1007/s00395-019-0742-1

**Published:** 2019-08-01

**Authors:** Mihai-Nicolae Podaru, Laura Fields, Satoshi Kainuma, Yuki Ichihara, Mohsin Hussain, Tomoya Ito, Kazuya Kobayashi, Anthony Mathur, Fulvio D’Acquisto, Fiona Lewis-McDougall, Ken Suzuki

**Affiliations:** William Harvey Research Institute, Barts and The London School of Medicine and Dentistry, Queen Mary University of London, Charterhouse Square, London, EC1M 6BQ UK

**Keywords:** Macrophages, Myocardial repair, Cell therapy, Myocardial infarction, Inflammation

## Abstract

Reparative macrophages play an important role in cardiac repair post-myocardial infarction (MI). Bone marrow mononuclear cells (BM-MNCs) have been investigated as a donor for cell therapy but with limited clinical success. These cells, however, may be utilized as a source for reparative macrophages. This translational study aimed to establish a robust in vitro protocol to produce functional reparative macrophages from BM-MNCs and to establish pre-clinical evidence of the efficacy of reparative macrophage transplantation for the treatment of MI. Mouse BM-MNCs were treated with M-CSF plus IL-4, IL-10, TGF-β1 or combinations of these in vitro. The concomitant administration of M-CSF and IL-4 produced the highest rate and largest number of CD11b^+^F4/80^+^CD206^+^ reparative macrophages. Expression and secretion of tissue repair-related factors including IGF-1, TGF-β1, VEGF and IL1-ra were remarkably enhanced in reparative macrophages compared to BM-MNCs. These cells were transplanted in a mouse MI model, resulting in evident improvement in cardiac function recovery, compared to BM-MNC transplantation. Histological studies showed that reparative macrophage transplantation enhanced myocardial tissue repair including augmented microvascular formation, reduced cardiomyocyte hypertrophy and attenuated interstitial fibrosis. Moreover, survival of reparative macrophages in the heart post-transplantation was increased compared to BM-MNCs. Reparative macrophage transplantation also increased host-derived reparative macrophages in part through TGF-β secretion. In conclusion, concomitant M-CSF + IL-4 treatment effectively produced reparative macrophages from BM-MNCs in vitro. Transplantation of produced reparative macrophage achieved a superior therapeutic efficacy, compared to BM-MNC transplantation, through the enhanced quantity and quality of donor cell engraftment. Further development of this advanced cell-based therapy is warranted.

**Electronic supplementary material:**

The online version of this article (10.1007/s00395-019-0742-1) contains supplementary material, which is available to authorized users.

## Introduction

Recent research has revealed that there are distinct subpopulations of macrophages in the heart, which contribute to the development of and recovery from myocardial damage post-MI in different ways [11, 21, 26]. Immediately after MI, there is a fierce inflammatory response mainly governed by infiltrating neutrophils, pro-inflammatory monocytes and M1-like macrophages [14]. The main aim of this response is to remove necrotic cell debris from the MI zone [14]. As such, in the first 3–4 days post-MI, the majority of macrophages in the heart exhibit pro-inflammatory M1-like phenotype, while this is followed by a quick increase of M2-like macrophages by days 5–7 [37]. M2-like macrophages are capable of secreting anti-inflammatory cytokines (e.g., IL-1ra, IL-10, TGF-β families), which contribute towards the resolution of inflammation in combination with pro-resolving lipid mediators [6]. M2-like macrophages also enhance formation of supportive connective tissues by activating fibroblasts through secretion of pro-fibrotic cytokines and by affecting the balance between matrix metalloproteinase (MMP) and tissue inhibitor of metalloproteinase (TIMPs) [29, 31, 32, 40]. Due to the inadequate regenerative ability of the heart, this process is crucial to prevent rupture or excessive dilatation of the fragile, infarcted ventricular walls. In addition, M2-like macrophages are involved in various stages of neovascular formation [28]. These data strongly suggest that further augmentation of M2-like macrophages will result in enhanced myocardial repair and improve prognosis post-MI.

Cell transplantation has recently attracted attention as a new therapeutic option for MI [1]. The ease of procurement of a large number of autologous cells, which include multiple types of stem/progenitor cells, has made bone marrow-derived mononuclear cells (BM-MNCs) an attractive target for investigation as a donor cell source. BM-MNCs have an ability for cardiac repair post-MI through their reparative secretome [13]. However, therapeutic efficacy of this approach in clinical trials has been inconsistent. Recent systematic reviews and meta-analyses provided evidence that the clinical benefit of this therapy is not as substantial as expected [4, 17]. The ongoing phase III clinical trial (BAMI) may conclude the efficacy of BM-MNC therapy [22].

Notably, BM-MNCs are a natural source of M2-like reparative macrophages. It is also possible to induce differentiation/polarization of BM-MNCs to M2-like macrophages in vitro using cytokines [2, 5, 16, 20, 33, 39]. Reparative macrophages are known to have the ability to secrete a powerful tissue-repairing secretome [41]. In addition, unlike BM-MNCs, which are foreign to the heart tissue, reparative macrophages are cells that naturally settle in the damaged heart, thereby possibly exhibiting improved survival in the heart. The innate ability of macrophages to migrate and settle into the damaged tissue may also help effective functional engraftment of transplanted cells in the damaged heart, compared to BM-MNCs [21]. We, therefore, hypothesize that directed differentiation of donor BM-MNCs to a reparative macrophage phenotype prior to transplantation would augment the efficacy of BM-MNC transplantation for the treatment of MI.

This research aimed to establish an effective, clinically applicable cytokine combination for obtaining BM-MNC-derived macrophages having tissue-repairing properties and furthermore investigated how transplantation of these reparative macrophages improved myocardial tissue repair and cardiac function in a mouse MI model.

## Materials and methods

The authors declare that all data which support the findings of this study are available within the article and its online supplementary files. Further information regarding this study is available from the corresponding author upon reasonable request.

### Animals

All animal studies were performed with the approval of the institutional ethics committee at Queen Mary University of London and the Home Office, UK. The investigation conforms to the Principles of Laboratory Animal Care formulated by the National Society for Medical Research and the Guide for the Care and Use of Laboratory Animals (US National Institutes of Health Publication, 1996). Male C57BL/6 mice, 8–10 weeks old (Charles River) were used in all in vitro experiments and for the generation of donor cells. The animals were culled by CO_2_ euthanasia immediately before the start of the bone marrow isolation procedure. For the in vivo experiments, the MI induction procedure was performed on 12-week-old female C57BL/6 mice (Charles River).

### Bone marrow extraction and BM-MNC isolation

The mouse bone marrow was harvested by flushing the hind limb tibia and femur with complete DMEM medium (DMEM containing 10% FBS, 1%PS; Gibco). The cell suspension was passed through a 70-µm cell strainer and was centrifuged for 5 min at 400*g*. The resulting cell pellet was washed twice with phosphate buffered saline (PBS) and then resuspended in 5 ml cold 1× red blood cell lysis buffer (BioLegend), shaking gently occasionally. The reaction was stopped by adding 20-ml PBS and the cells were pelleted (5 min at 350*g*). Cells were resuspended in PBS, loaded onto Histopaque©-1077 (Sigma-Aldrich) and centrifuged for 30 min at 400*g* without brake. The interphase (containing BM-MNC) was carefully collected, washed once with PBS and twice with medium according to the manufacturer’s instructions. The cells were then resuspended and counted.

### M2-like macrophage generation protocol

Freshly isolated BM-MNCs were seeded onto Nunc© Cell Culture Treated Flasks with Filter Caps (ThermoFisher) at a concentration of 3.6 × 10^4^ cells/cm^2^. The cells were treated by supplementing complete DMEM with M-CSF (20 ng/ml; Peprotech) and either IL-4, IL-10, TGF-β1 (20 ng/ml; Peprotech) or specific combinations of them. These cytokines were chosen based on the fact that they have been extensively studied and their efficacy for M2-macrophage polarisation was well characterised in previous publications [2, 5, 16, 20, 33, 39]. Specifically, the following treatments were analyzed: M-CSF + IL-4; M-CSF + IL-10; M-CSF + IL-4 + IL-10; M-CSF + IL-4 + TGF-β1; M-CSF + IL-4 + IL-10 + TGF-β1. Freshly isolated BM–MNC (untreated) and M0 unpolarized macrophages (M-CSF only treated) were used as controls. The medium was changed at day 4 with fresh cytokines.

Alternatively, M2-like macrophages were produced using a sequential treatment protocol involving the initial treatment of BM-MNC with M-CSF only (20 ng/ml) for 5 days, followed by treatment with IL-4 only (20 ng/ml) for an additional day (6 day protocol). M1-like macrophages were generated by treating BM-MNC with M-CSF (20 ng/ml) for 5 days and after that by IFN-γ (20 ng/ml) and LPS (50 ng/ml) for another 2 days. M-CSF was withdrawn from the culture medium in the M1 polarization step.

### Flow cytometry

Cells were collected from the culture flasks by scraping, counted and resuspended in PBS to yield 4–5 × 10^5^ cells/tube (minimum 2 × 10^5^). Cells were pelleted (centrifuged at 300*g* for 5 min) and resuspended in 100-µl flow cytometry buffer (5% FBS, 0.002% NaN_3_ in PBS). The cells were blocked with anti-mouse CD16/32 antibody (IgG2a, 93, monoclonal, rat; 1:100) for 30 min on ice, and then incubated with conjugated antibodies (Table S1) for 30 min on ice. Samples stained with suitable IgG controls (Table S1) served as negative controls and were also used for gating purposes. Finally, the cells were washed once with buffer, resuspended in 500-µl buffer, and further stained with DAPI (2 ng/µl) as a viability marker and transferred to polystyrene flow cytometry tubes. Expression of macrophage surface markers was assessed using the BD LSRFortessa© cell analyzer and the acquired data were further processed with the FlowJo software (v.10). In each sample, 10,000 events in the final gate were recorded. Appropriate compensation was performed using UltraComp© eBeads (Invitrogen) before each experiment. Cellular debris, doublets and dead cells were excluded during the processing step (Figure S1).

### RNA extraction from cultured cells

Macrophages and BM-MNCs (1.5 × 10^6^ cells/sample) were collected from culture by scraping. RNA was extracted using TRIzol© reagent (Invitrogen) according to the manufacturer’s instructions. The RNase-Free DNase set (Qiagen) was used to digest contaminating DNA according to the manufacturer’s instructions. The purified RNA was either used immediately for downstream applications or stored at − 20 °C for short-term use.

### cDNA reverse transcription

Reverse transcription of RNA samples was carried out using the Applied Biosystems High-Capacity cDNA Reverse Transcription Kit© according to manufacturer’s instructions. Briefly, 2× reverse transcription (RT) master mix was prepared using the reagents provided in the kit. Then, 1 µg/ml RNA in a 10-µl volume was added to 10 µl of 2× Master Mix or to Master Mix without the reverse transcriptase (no RT control). In addition, a no-template control was prepared (master mix with no sample added). All these steps were performed on ice. All the resulting samples were briefly centrifuged and loaded into a thermal cycler.

### Real-time qPCR

PCR primers (IDT) for the following genes: *Arg1, Fizz1, Ym1, Tnfa, Il*-*10, Igf1, Tgfb1, Vegfa*, *IL*-*1ra, Mmp9, Ptges2, Timp1 and Spp1* were used (Table S2). The analysis was carried out on freshly isolated BM-MNC (control), M2(IL-4) macrophages and inflammation-subjected M2(IL-4) macrophages. *GAPDH* was used as an internal control for normalizing relative expression levels between samples. Real-time quantitative PCR was performed from reverse transcribed cDNA samples using the Powerup SYBR Green Master Mix. Briefly, 5 ng of cDNA was added to a MicroAmp© Optical 96-well reaction plate (Applied Biosystems) with 1× Powerup SYBR Green© Master Mix, 2.4 μl of nuclease free water and forward and reverse primers (both at 10 µM). Thermal cycling and fluorescent monitoring was performed using the StepOne© Detection System (Applied Biosystems). For each target gene, besides the biological replicates, three technical replicates were performed. Negative controls using RNA as template were also included in all runs to test for the possible genomic DNA contamination of the samples.

### Cell yield calculations

The number of cells was recorded at the start and the end of each protocol by trypan blue dye (0.4%, ThermoFisher Scientific) staining and automated cell counting (Countess© II Automated Cell Counter, Thermo Fisher). The yield was calculated using the following formula: Yield (%) = number of viable harvested cells/starting viable cell number × 100.

### Inflammatory stimuli treatment of M2(IL-4) macrophages

M2-like macrophages (M-CSF + IL-4 polarized, at day 6 of the protocol) were subjected to inflammatory stimuli (IFN-γ-20 ng/ml and LPS 50 ng/ml; Peprotech) for 6 h in complete medium [24]. Subsequently, the inflammatory stimuli were withdrawn and the macrophages were used in downstream applications: qPCR analysis or ELISA.

### Conditioned medium experiments

M2(IL-4) macrophages or freshly isolated BM-MNCs were incubated for 24 h with serum-free DMEM. After the culture period, the medium was collected and centrifuged at 300*g* for 5 min and the supernatant was used in further applications as M2(IL-4) macrophage or BM-MNC-conditioned medium. M0 (unpolarised) macrophages were obtained by treating freshly isolated BM-MNCs with M-CSF (20 ng/ml) for 5 days. These cells were then incubated for 24 h with conditioned medium from BM-MNC or M2(IL-4) macrophages. In addition, either 50 μg/ml TGF-β pan-specific neutralizing antibody (R&D Systems) [19] or equal amount of rabbit polyclonal antibody was applied to the M2(IL-4)-conditioned medium. M-CSF (20 ng/ml) was maintained for the co-culture duration.

### Enzyme linked immunosorbent assay (ELISA)

ELISA was performed on conditioned medium from freshly isolated BM-MNC and IL-4-polarized M2 macrophages. Regardless of the condition, the cells received fresh medium without any cytokines and were maintained in culture for 24 h. The medium was then collected and analyzed using ELISA kits (Quantikine© ELISA kits for TGF-β1, VEGF, IL-1ra and IGF-1, R&D Systems) according to the manufacturer’s instructions.

The IGF-1 samples required a 1:2 dilution, while the TGF-β1 samples required activation with 1 N HCl and 1.2 N NaOH/0.5 M HEPES in dH_2_O before the start of the protocol.

### PKH26 staining

Both BM-MNC and M2(IL-4) macrophages were stained with PKH26 ethanoic dye solution (1 mM, Sigma Aldrich) immediately prior to transplantation into animals, according to the manufacturer’s instructions. The PKH26 dye was diluted (to 4 μM) in diluent C and 25 μl of this solution was used per 5 × 10^5^ harvested cells (1 × 10^7^ cells/ml). The diluted dye was immediately added to an adequate equal volume of diluent C that was already containing the cell suspension and left to incubate for 4 min. Homogenous staining was achieved by vigorous mixing. Finally, an equal volume of 100% FBS was added to the solution to stop the staining reaction. The cells were then washed three times in 10-ml complete DMEM medium. Successful staining was assessed immediately after staining by bright field microscopy using a BZ800 Keyence Microscope.

### Myocardial infarction induction and cell transplantation

As previously described [30], MI was induced in 12-week-old female C57BL/6 mice by ligation of the left coronary artery using an 8–0 polypropylene suture through left thoracotomy under 2.0% isoflurane anesthesia and mechanical ventilation (intubation aided by tracheotomy). The success of the procedure was determined by the positive observation of myocardial discoloration and changes in the left ventricular wall mechanics. Immediately after ligation, two injections of 10-μl PBS each containing male, PKH26 pre-labelled cells (5 × 10^5^ BM-MNC or 5 × 10^5^ M2-IL-4 macrophages in total) or PBS alone were injected intramyocardially in the infarct border zone. The chest and skin were then closed using a 5–0 VicrylPlus© Antibacterial suture. The animals were placed on a heating pad and carefully observed for the next 2–3 h until they achieved full locomotor behavior. Analgesics (0.5 mg/kg—subcutaneous injection) were administered before the surgery and then twice a day for a duration of 3 days after surgery.

### Echocardiography

Transthoracic echocardiography was performed 28 days after treatment using a Vevo-3100 Preclinical Imaging Platform (VisualSonics© Fuji Film) with a 70-MHz ultrahigh-frequency transducer under 2.0% isoflurane inhalation [30]. Briefly, the mice were abdominally shaved, anesthetized and placed on a heat pad containing electrocardiography electrodes and capable of tracing heart rate, respiratory rate and internal temperature. Systolic (LVDs) and diastolic (LVDd) left ventricular dimensions as well as fractional shortening were measured from the short axis M-mode images using the VevoLab (version 3.1.0) software. Data were collected from three different measurements made on three different echocardiographic images per mouse in a blinded manner.

### Quantitative assessment of donor cell presence and RNA extraction from left ventricular tissue

To quantify the presence of engrafted male, mouse BM-MNC and M2(IL-4) macrophages in the female mouse heart, the presence of the Y chromosome-specific *Sry* gene was quantitatively assessed by real-time PCR as previously described [12]. The left ventricular myocardium was collected 28 days after treatment (with removal of atriums and right ventricular free walls) and preserved in RNAlater (Ambion). The tissue was homogenised using the Percellys Lysing kit containing ceramic beads. The resulting lysate was further disrupted using QIAshredder (Qiagen). Genomic DNA was extracted using the AllPrep DNA/RNA Mini Kit according to the manufacturer’s instructions. To generate a standard curve, left ventricular myocardium from female mice was mixed with known amounts of male mouse M2(IL-4) macrophages or BM-MNCs and subsequently processed for *Sry* analysis. The signal in each LV sample was normalized to the amount of DNA by measuring the autosomal single-copy gene *GAPDH* as an internal standard and read against the standard curve.

RNA was also extracted from the same heart samples using the AllPrep DNA/RNA Mini Kit according to the manufacturer’s instructions. Genomic DNA digestion was performed using the RNase-Free DNase set (Qiagen) according to the manufacturer’s instructions. The RNA was immediately used in downstream applications or stored at − 20 °C until required.

### Cryosectioning

Mice were culled by CO_2_ inhalation and their hearts were immediately excised, rinsed in PBS, frozen in optimal cutting temperature (OCT) compound and stored at − 80 °C. Hearts were serially cryosectioned at a thickness of 6 μm using a Bright cryostat. The cryosections were then transferred on Polysine Adhesion Slides (Thermo Scientific) and maintained at − 80 °C until required for staining.

### Immunohistochemistry and histological analysis

The cryosections were thawed and fixed for 30 min with 4% paraformaldehyde, washed three times in PBS (5 min each), permeabilized in Triton X-100 (0.1%) for 10 min and washed again three times in PBS. This was followed by blocking with 5% BSA/PBS solution for 1 h. Subsequently, the cells were incubated with primary antibodies (Table S3) overnight at 4 °C, washed three times in PBS and afterwards incubated with the secondary antibodies (Table S3) for 1 h at room temperature. Conjugated WGA-FITC was added together with the secondary AF647 antibody (for 1 h). All antibodies were diluted to the working concentration in 5% BSA/PBS solution. Sections were further stained with DAPI (1 ng/µl). Finally, the sections were washed again three times in PBS and the slides were mounted with Dako Mounting Medium (Agilent).

Images were captured using a fluorescence microscope (BZ8000; Keyence, Milton Keynes, UK; 20× or 40× objectives) and analyzed using ImageJ software. The quantitative assessments of CD206^+^ M2-like macrophages and blood capillary density (IB4 staining) were conducted in five randomly selected fields per each area of the heart (infarct, border, remote). To evaluate cardiomyocyte size, the cross-sectional area of appropriately detected α-sarcomeric actinin^+^ cardiomyocytes (transversely cut, exhibiting central nuclei) was measured for 40 cardiomyocytes from 5 fields of view per area (border and remote). PKH26 and CD206 co-staining was assessed from four fields of view per area.

### Picrosirius red staining

Frozen heart sections were fixed in 4% paraformaldehyde, incubated in 0.1% Picrosirius red solution for 60 min and then in 0.5% of acetic acid solution for 3 min. Sections were washed three times for 5 min between each of these steps. The samples were dehydrated through immersion in increasing concentrations of ethanol (70% and 100%) and xylene (20 s each immersion). Finally, the sections were mounted using DPX mounting medium (VWR International). The wall thickness was measured at five independent regions of the infarct area. The quantity of the collagen fraction was calculated from five fields (20× magnification) of each area per heart using the ImageJ software [30]. In addition, infarct size was measured as the ratio of both epicardial and endocardial scar lengths relative to total epicardial and endocardial circumference as previously described [35].

### Statistical analysis

The statistical analysis was conducted using the GraphPad Prism (v.5.04) software. All data sets were statistically analyzed by either performing the one-way ANOVA analysis followed by Bonferroni’s Multiple Comparisons Test (three or more sets of data) or Student’s *t* test (two sets of data). All data sets are presented as mean ± SEM. The significance threshold was set at *p* < 0.05.

## Results

### M-CSF + IL-4 treatment was effective to produce M2-like macrophages

**Fig. 1:**
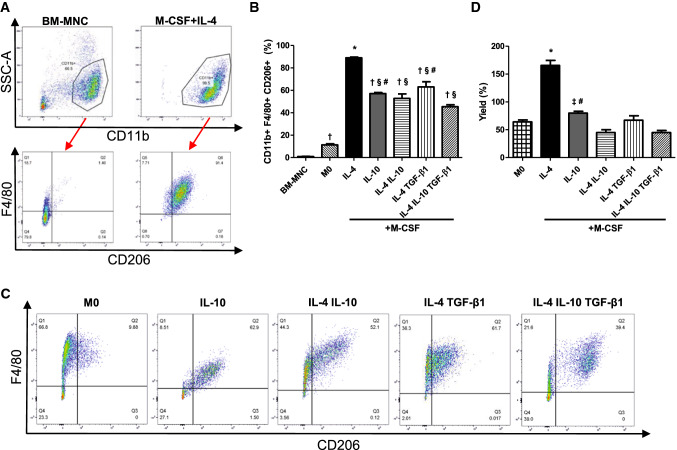
Production of M2-like macrophages by M-CSF + IL-4 treatment. Mouse BM-MNCs were isolated and treated with different M2-like macrophage induction protocols (M-CSF plus combination of polarizing cytokines) for 7 days. Freshly isolated BM-MNCs and M0 macrophages (treated with M-CSF alone) were used as controls. Bar charts presented as mean ± SEM. **a** Gating strategy for flow cytometry procedures. Cells double positive for F4/80 and CD206 within the CD11b^+^ population were defined as M2-like macrophages by flow cytometry. Representative dot plots from samples of freshly isolated BM-MNCs and M-CSF + IL-4 treated BM-MNCs are presented. **b** Quantification of M2-like macrophage induction. Percentages of M2-like macrophages among total cultured cells were measured by flow cytometry at the end of the induction protocols. *p < 0.05 vs. all other groups; ^†^*p* < 0.05 vs. BM-MNC; ^§^*p* < 0.05 vs. M0; ^#^*p* < 0.05 vs. IL-4 IL-10 TGF-β1. *N* = 4–7 independent samples. **c** Representative flow cytometry images for each induction protocol. Representative dot plots from M0; IL-10; IL-4 + IL-10; IL-4 + TGF-β1; IL-4 + IL-10 + TGF-β1 protocols are presented. **d** Quantification of M2-like macrophage yield. For each protocol, the yield after 7 days of culture was defined as the obtained number of M2-like macrophages in relation to the starting number of BM-MNCs and expressed as a percentage. *p < 0.05 vs. all other groups; ^‡^*p* < 0.05 vs. IL-4 + IL-10; ^#^*p* < 0.05 vs. IL-4 + IL-10 + TGF-β1. *N *= 4–11 independent experiments

To optimize the in vitro treatment to produce M2-like macrophages, mouse BM-MNCs were treated with M-CSF and one or combinations of known M2-like polarizing cytokines, including IL-4, IL-10 and TGF-β1 for 7 days. Approximately, 70% of freshly isolated BM-MNCs were positive for CD11b, a monocyte precursor marker (Figs. 1a, S2). However, triple positive cells for CD11b, F4/80 and CD206, which were defined as M2-like macrophages [31], were hardly found in BM-MNCs (Fig. 1a, b). In contrast, after 7 days of in vitro culture with M-CSF + IL-4, over 99% of the cells were positive for CD11b (Figure S2) and approximately 90% of these cells were also positive for F4/80 and CD206 (Fig. 1b, c). Culture with M-CSF + IL-10 achieved only 60% triple positivity, while M-CSF + TGF-β1 did not allow BM-MNCs to survive for 7 days. Addition of TGF-β1 and/or IL-10 to the M-CSF + IL-4 treatment reduced production of CD11b^+^ F4/80^+^CD206^+^ M2-like macrophages (Fig. 1b, c). Furthermore, the absolute number of produced CD11b^+^F4/80^+^CD206^+^ M2-like macrophages was increased by M-CSF + IL-4 treatment compared to other treatments (Fig. 1d). The M-CSF + IL-4 treatment increased by approximately 1.7-fold the number of CD11b^+^F4/80^+^CD206^+^ M2-like macrophages as compared to the originally plated BM-MNCs.

**Fig. 2:**
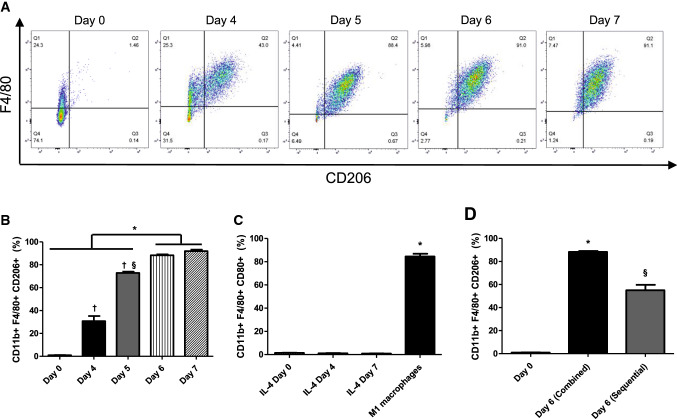
Optimization of M-CSF + IL-4 treatment. The M-CSF + IL-4 treatment was further analyzed in terms of duration, purity and treatment sequence. Bar charts presented as mean ± SEM. **a** Representative images for the timeline of M2-polarization by M-CSF + IL-4 treatment. BM-MNCs were treated with M-CSF + IL-4 and analyzed for M2-like macrophage markers by flow cytometry at days 4, 5, 6 and 7. Freshly isolated BM-MNCs (day 0) served as controls. **b** M2-like macrophage polarization timeline by M-CSF + IL-4 treatment. Percentages of M2-like macrophages among total cultured cell numbers were calculated from flow cytometry (refer to **a**). *both days 6 and 7 significant *p* < 0.05 vs. all days 0, 4 and 5, ^†^*p* < 0.05 vs. day 0; ^§^*p* < 0.05 vs. day 4. *N* = 4–6 independent samples. **c** M1-like macrophage production by M-CSF + IL-4 treatment. BM-MNCs were treated with M-CSF + IL-4 and analyzed for M1-like macrophage markers (CD80^+^) by flow cytometry at days 0, 4 and 7. M1-like macrophages were obtained by treatment of BM-MNCs with M-CSF, LPS and IFN-γ and served as positive controls. *p < 0.05. *N* = 4 independent samples. **d** Comparison against the sequential polarization approach. BM-MNC were either cultured concomitantly with M-CSF and IL-4 for 6 days (combined) or initially with M-CSF for 5 days and polarized with IL-4 only for 1 further day (sequential) and analyzed for M2-like macrophage markers by flow cytometry. *p < 0.05 vs both day 6 (sequential) and day 0; ^§^*p* < 0.05 vs day. *N* = 4–6 independent samples

As M-CSF + IL-4 treatment was the most effective in producing CD11b^+^F4/80^+^CD206^+^ M2-like macrophages, we aimed to determine its minimal required culture period. Flow cytometry analysis clarified that the percentage of CD11b^+^F4/80^+^CD206^+^ cells started increasing by day 4 and plateaued from day 6 at around 90% (Fig. 2a, b). In addition, these cells started to show specific M2-like morphologic characteristics [23] as early as day 4 but with larger numbers of cells exhibiting the spindle-like shape morphology from day 5 (Figure S3). In addition, the percentage of triple positive cells for CD11b, F4/80 and CD80 was below 1.5% at all times points tested (Fig. 2c), suggesting that contamination with M1-like pro-inflammatory macrophages was negligible. Taken these data together, a 6-day duration of the M-CSF + IL-4 treatment was sufficient to produce exclusively M2-like macrophages from BM-MNCs.

Previous studies have reported that culture with M-CSF and IL-4 favored M2-like macrophage differentiation/polarization of BM-MNCs in rodents [2, 5, 16, 20, 33, 39]. However, each of these earlier reports used a sequential methodology, i.e., initial culture with M-CSF (5–7 days) followed by additional culture in the presence of IL-4 (24–48 h without M-CSF). We, thus, compared the efficacy to produce CD11b^+^F4/80^+^CD206^+^ M2-like macrophages from BM-MNCs between this sequential method (5-day M-CSF treatment followed by 24 h of IL-4 treatment) and our treatment (concomitant addition of both M-CSF and IL-4 for 6 days). As a result, it was demonstrated that the concomitant M-CSF + IL-4 treatment led to a higher percentage of CD11b^+^F4/80^+^CD206^+^ M2-like macrophages compared to the sequential approach (Fig. 2d). Following recommendations [27], cells produced using the M-CSF + IL-4 concomitant treatment were named M2(IL-4) macrophages.

### M2(IL-4) macrophages exhibited a reparative phenotype

**Fig. 3:**
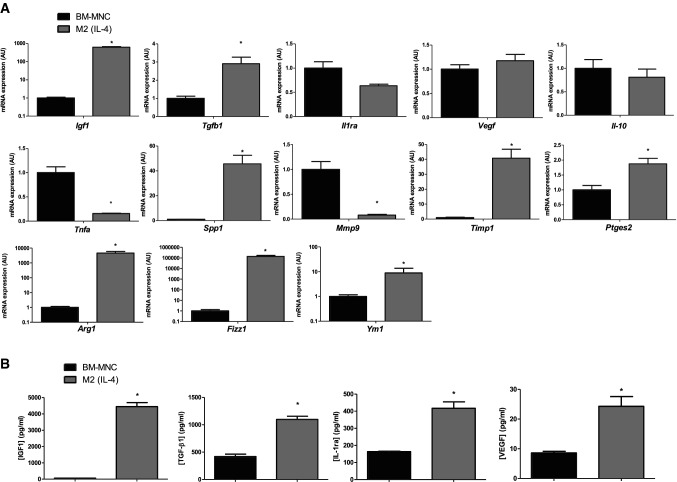
Pro-reparative, anti-inflammatory phenotype of M2(IL-4) macrophages. Gene expression and cytokine secretion profiles of M2-like macrophages obtained after 6 days of M-CSF + IL-4 treatment [M2(IL-4) macrophages] and freshly isolated BM-MNCs were compared. Bar charts presented as mean ± SEM. **a** Upregulation of M2-like macrophage markers and myocardial repair-related genes in M2(IL-4) macrophages. Fold change in mRNA levels were assessed by real-time qPCR using *GAPDH* as a reference gene. Expression in BM-MNCs was normalized to 1, which denotes no change in mRNA relative expression normalized to *GAPDH*. Data pooled from 6 to 12 independent biological samples and three technical replicates. *p < 0.05. **b** Pro-reparative, anti-inflammatory secretome of M2(IL-4) macrophages. Cytokines levels were assessed by ELISA after 24 h of culture. Data pooled from three independent biological samples and two technical replicates. *p < 0.05

To examine the potential ability to promote cardiac repair, the gene and protein expression profile of M2(IL-4) macrophages was analyzed in comparison to BM-MNCs. M2(IL-4) macrophages showed significant upregulation of M2-marker genes including *Arg1, Fizz1* and *Ym1,* as well as genes reported to have a potential ability of tissue healing and repair post-MI, including *Igf1, Tgfb1, Spp1,* and *Ptges2* [15, 25, 29, 31, 32, 40] (Fig. 3a). *Mmp9* was downregulated, while the gene expression of *Il1ra*, *Vegf* and *Il*-*10* was not significantly different between the two cell types. In addition, *Tnfa* was downregulated in M2(IL-4) macrophages, highlighting the anti-inflammatory profile of these cells. These observations were further investigated in terms of protein secretion (Fig. 3b). Substantial increases in IGF-1, TGF-β1, IL-1ra and VEGF protein secretion were observed in the M2(IL-4) macrophage culture medium when compared to BM-MNCs. These data provided evidence that M2(IL-4) macrophages have a superior potential of secreting anti-inflammatory and pro-reparative factors. To induce cardiac tissue repair post-MI in vivo, it is important that M2(IL-4) macrophages maintain their reparative phenotype in an inflammatory and ischemic environment. Thus, we investigated a possible re-polarization of M2-like macrophages to an M1-like phenotype, by exposing M2(IL-4) macrophages to LPS and IFN-γ stimulation in vitro. As a result, it was shown that inflammatory stimulation did not negatively affect expression of tissue repair-related and M2-like marker genes, suggesting that M2(IL-4) macrophages could maintain their M2-like reparative phenotypes even after transplantation into the heart post-MI (Figure S4).

### M2(IL-4) macrophage transplantation showed augmented therapeutic effects in a mouse MI model

**Fig. 4:**
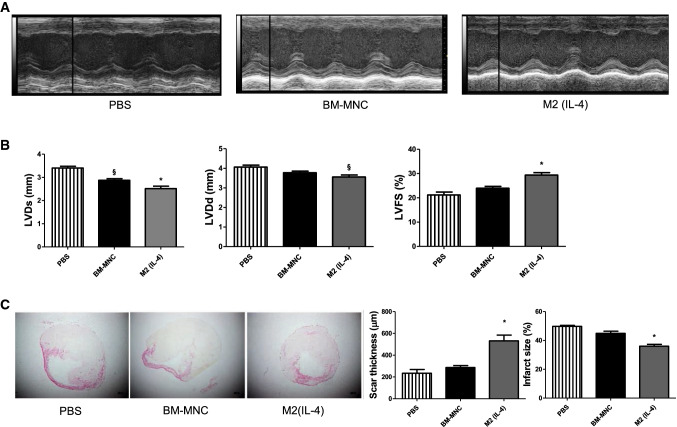
Enhanced post-MI cardiac recovery by M2(IL-4) macrophage transplantation. After induction of MI by left coronary artery ligation in mice, 5 × 10^5^ BM-MNCs or 5 × 10^5^ M2(IL-4) macrophages or PBS alone were administered intramyocardially to the border zone surrounding the infarct. Bar charts presented as mean ± SEM. **a** Representative echocardiography images at day 28 after treatment. M-mode short axis echocardiography images highlighting changes in anterior wall motion in relation to treatment. **b** Improved cardiac function and reduced ventricular dimension by M2(IL-4) macrophage transplantation. LVDs (left ventricular dimension at systole), LVDd (left ventricular dimension at diastole) and LVFS (left ventricular fractional shortening) were measured using transthoracic echocardiography (see **a**). *p < 0.05 vs. both BM-MNC and PBS groups; ^§^*p* < 0.05 vs. PBS group. *N* = 9 mice in PBS and BM-MNC groups and *N* = 11 mice in M2(IL-4) group. **c** Increased scar thickness and reduced infarct size by M2(IL-4) macrophage transplantation. Hearts were collected at day 28 after treatment and subjected to picrosirius red staining. Representative images of each group are presented. Thickness of infarct left ventricular wall and infarct size were measured using these samples. *p < 0.05 vs. both BM-MNC and PBS groups. *N* = 4 hearts in PBS and BM-MNC groups and *N* = 5 hearts in M2(IL-4) group

Although the aforementioned data suggested that M2(IL-4) macrophages could be a suitable donor cell type for MI therapy, it was still to be determined whether transplantation of these cells could augment improvements in cardiac function in vivo. To this end, after MI induction by left coronary artery ligation, mice received intramyocardial injection of either (i) 5 × 10^5^ M2(IL-4) macrophages produced from syngeneic mouse BM-MNCs using the above optimized M-CSF + IL-4 treatment, (ii) 5 × 10^5^ freshly isolated syngeneic mouse BM-MNCs or (iii) PBS control. At 28 days after cell transplantation, echocardiography demonstrated that M2(IL-4) macrophage transplantation exhibited an increase in left ventricular fractional shortening (LVFS) when compared to both BM-MNC and PBS control groups (29.4 ± 1.0%, 24.0 ± 0.8%, 21.2 ± 1.2%, respectively; Figs. 4a, b). Thus, LVFS after M2(IL-4) macrophage transplantation was increased by approximately 8% compared to the PBS control group. BM-MNC transplantation showed a tendency of improvement of LVFS compared to the control but failed to achieve statistical significance. Both end-systolic and diastolic LV dimensions were improved by the M2(IL-4) macrophage transplantation when compared to both PBS control and BM-MNC treatment. BM-MNC transplantation only achieved significance in reducing the systolic LV dimension. Furthermore, histological analysis revealed that M2(IL-4) macrophage transplantation reduced the infarct size with increased thickness of the infarcted LV free wall compared to both PBS control and BM-MNC treatment (Fig. 4c). The mortality rates were comparable among the groups studied. The PBS control group and the BM-MNC transplantation group showed a mortality rate of 18.2% (2 out 11 mice), while the M2(IL-4) group exhibited a mortality rate of 21.4% (3 out of 14 mice).

### M2(IL-4) macrophages exhibited enhanced engraftment post-transplantation

**Fig. 5:**
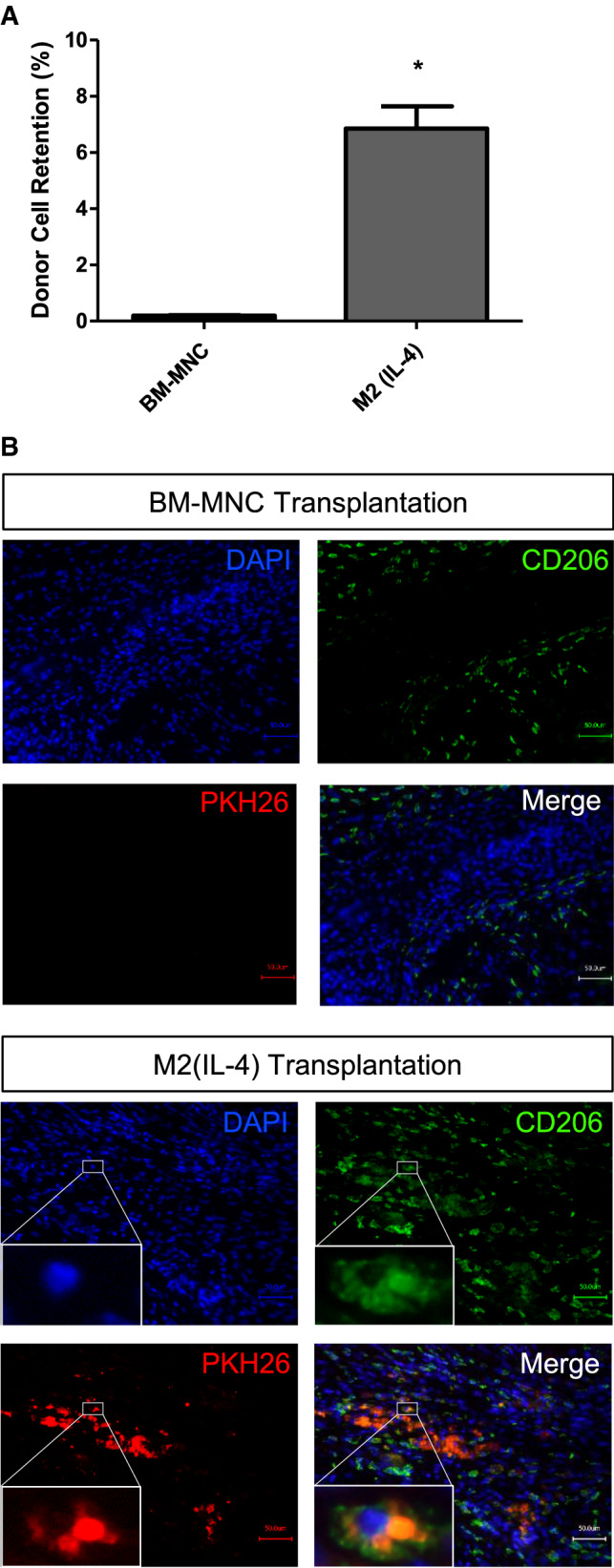
Enhanced donor cell survival after M2(IL-4) macrophage transplantation. Donor cell retention and localization was analyzed 28 days post-transplantation. Bar charts presented as mean ± SEM. **a** Quantitative donor cell retention. Quantitative cell retention of male BM-MNCs or M2(IL-4) macrophages in the female heart (LV wall excluding the ventricular septum) was carried out by analyzing genomic DNA levels of male-specific *Sry* gene within the left ventricular myocardium by qPCR. *Sry* signal was normalized for *GAPDH* expression and the corresponding number of cells was determined. Data presented as percentage of total transplanted cells. *N *= 5 hearts in each group; *p < 0.05. **b** Donor cell localization. Immunohistolabeling for CD206 and DAPI were performed using heart samples collected at day 28 after treatment. Donor cells were pre-labelled with PKH26 before transplantation. Representative images from the infarct area of the M2(IL-4) macrophage transplantation group are presented (lower lane). PKH26-positive donor cells were not detected in the BM-MNC group (upper lane). Scale bars = 50 μm

To investigate the factors underpinning the enhanced therapeutic efficacy of M2(IL-4) macrophage transplantation, the engraftment capacity was assessed by quantitative PCR for male-specific *Sry* gene in the LV myocardial samples. As the donor cells were of male origin and the recipient mice were females, this method enabled a robust quantitative estimation of the donor cell number existing in the heart [12]. The result elucidated a remarkable (> 30-fold) increase in donor cell engraftment for the M2(IL-4) macrophage transplantation (Fig. 5a). At 28 days after cell transplantation, 6.9 ± 0.8% of the total transplanted donor M2(IL-4) macrophages were still present in the LV in comparison to only 0.2 ± 0.0% for BM-MNCs. This finding was further validated by immunofluorescence where a sizable number of PKH26 pre-labelled donor cells were identified in the myocardium post-M2(IL-4) macrophage transplantation, while no PKH26-labelled cells were found in the BM-MNC treated hearts (Fig. 5b). In addition, transplanted M2(IL-4) macrophages persistently expressed CD206 in vivo even at 28 days post-transplantation, suggesting that the donor M2(IL-4) macrophages could maintain their reparative M2-like phenotype despite the hazardous, inflammatory environment post-MI (Fig. 5b). Of note is that donor cell-derived M2(IL-4) macrophages were surrounded by a number of PKH26-negative CD206-positive cells, i.e., host-derived M2-like macrophages.

### M2(IL-4) macrophage transplantation improved myocardial tissue repair post-MI

**Fig. 6:**
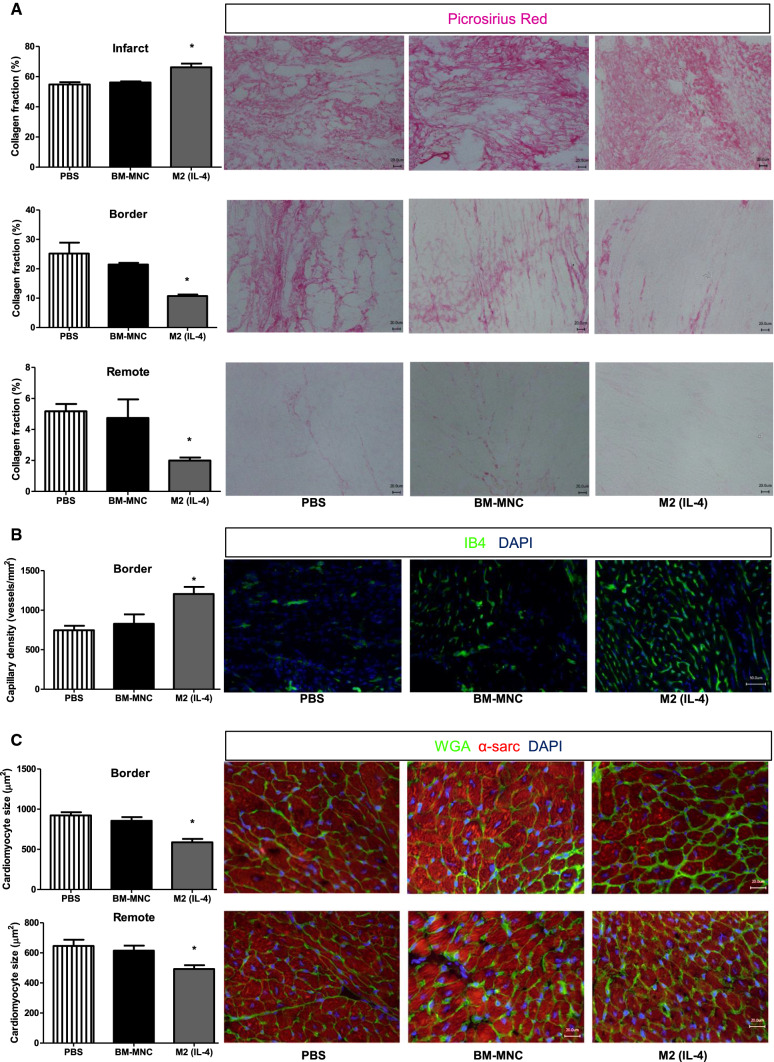
Improved myocardial tissue repair after M2(IL-4) macrophage transplantation. The hearts were collected 28 days after treatment and analyzed for myocardial tissue repair by histological assessments. Bar charts presented as mean ± SEM. *p < 0.05 vs. both PBS and BM-MNC groups; *N* = 4 hearts in PBS and BM-MNC groups and *N* = 5 hearts in M2(IL-4) group. **a** Picrosirius red staining for quantification of interstitial fibrosis. Collagen volume fraction was measured in the infarct, infarct border zone and remote myocardium. Scale bars = 20 μm. **b** Isolectin-B4 (IB4) staining for quantification of microvascular formation in the border area. The number of IB4-positive capillaries was counted and presented as vessels/mm^2^. Scale bars = 50 μm. **c** Wheat germ agglutinin (WGA) and α-sarcomeric actinin (α-sarc) staining to measure cardiomyocyte size. The cross-sectional area of cardiomyocytes (α-sarc^+^, transversely cut, central nuclei) in border and remote areas was measured. At least 40 cardiomyocytes per area were assessed. Scale bars = 20 μm

Various histological analyses revealed that the cardiac functional improvement induced by M2(IL-4) macrophage transplantation was underpinned by enhanced post-MI myocardium repair. Picrosirius red staining demonstrated that M2(IL-4) macrophage therapy facilitated denser collagen network formation (replacement fibrosis) in the infarct area (Fig. 6a), compared to both BM-MNC transplantation and PBS control group. This would confer stability of infarcted ventricular wall against the risk of excessive dilatation as observed in Fig. 4. On the other hand, pathological interstitial fibrosis in the viable myocardium of the remote and border areas was attenuated in the M2(IL-4) macrophage-treated hearts compared to the other groups (Fig. 6a). Although BM-MNC transplantation appeared to reduce interstitial fibrosis in the border area compared to the control group, this trend was not significant. Isolectin B4 staining for blood capillaries elucidated that there was an increased capillary presence in the border area of M2(IL-4) macrophage-treated hearts compared to other groups (Fig. 6b). The same observation held true for the infarct area, while there was no difference in the remote zones where the number of capillaries in all groups fell within normal ranges (Figure S5). Wheat germ agglutinin and α-sarcomeric actinin staining showed that the M2(IL-4) macrophage transplantation reduced the size of surviving cardiomyocytes in both border and remote areas of the heart compared to both other groups (Fig. 6c).

### M2(IL-4) macrophage transplantation improved myocardial expression of reparative genes

**Fig. 7:**
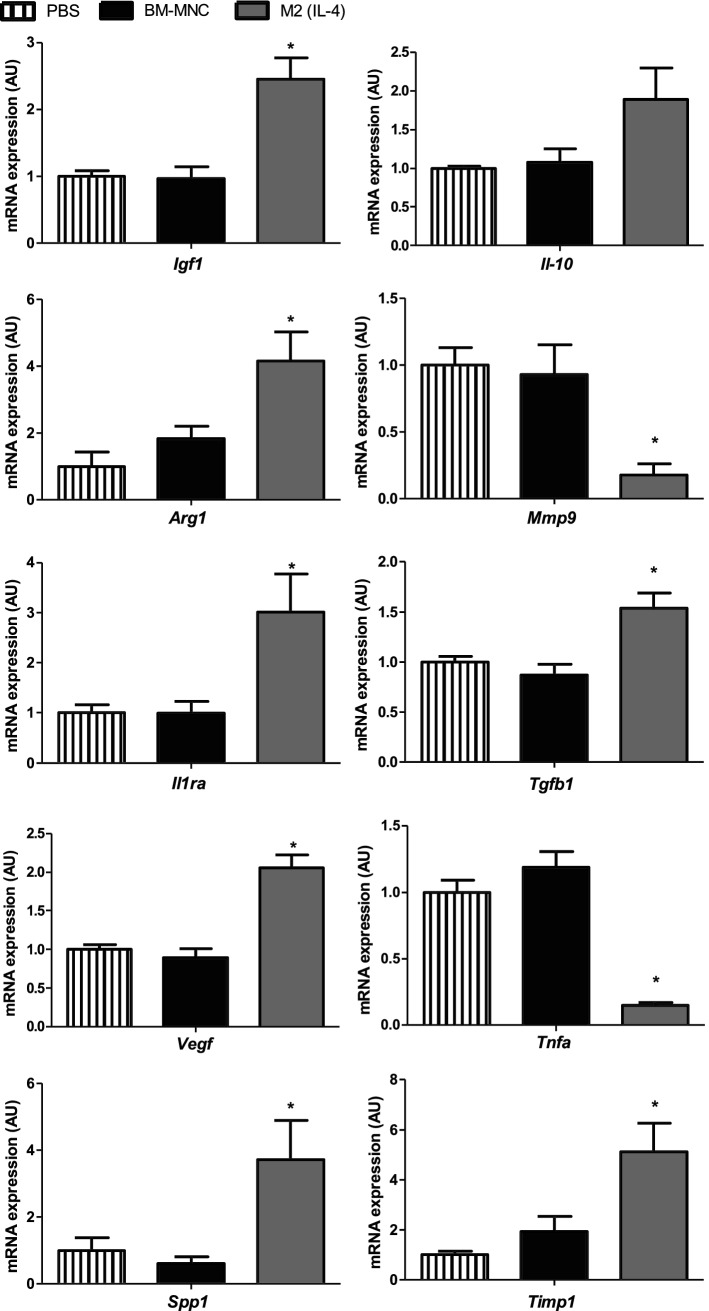
Upregulation of tissue repair-related genes in the myocardium after M2(IL-4) macrophage transplantation. Fold change in mRNA levels for myocardial repair-related factors were assessed in the left ventricular myocardium (excluding the ventricular septum) collected at day 28 after treatment using real-time qPCR with *GAPDH* as a reference gene. Expression in PBS treated hearts was normalized to 1, which denotes no change in mRNA relative expression normalized to *GAPDH*. Data pooled from five independent biological samples in each group and three technical replicates. Bar charts presented as mean ± SEM. *p < 0.05 vs. both PBS and BM-MNC groups

In support of the histological findings of myocardial tissue repair, real-time quantitative PCR identified upregulation of a group of tissue repair-related factors in the M2(IL-4) macrophage-treated hearts. There was upregulation of *Igf1, Arg1, Il1ra. Tgfb1, Vegf, Tnfa, Spp1, Timp1* compared to both BM-MNC transplantation and PBS control groups, while *Mmp9* and *Tnfa* were downregulated (Fig. 7). *Il*-*10* gene showed no significant change in expression among all groups studied. Of note, the profiles of these upregulated reparative and inflammation-related genes in vivo were consistent with the findings of gene upregulation/downregulation of M2(IL-4) macrophages we observed in vitro (Fig. 3a).

### M2(IL-4) macrophage transplantation augmented endogenous M2-like macrophages

**Fig. 8:**
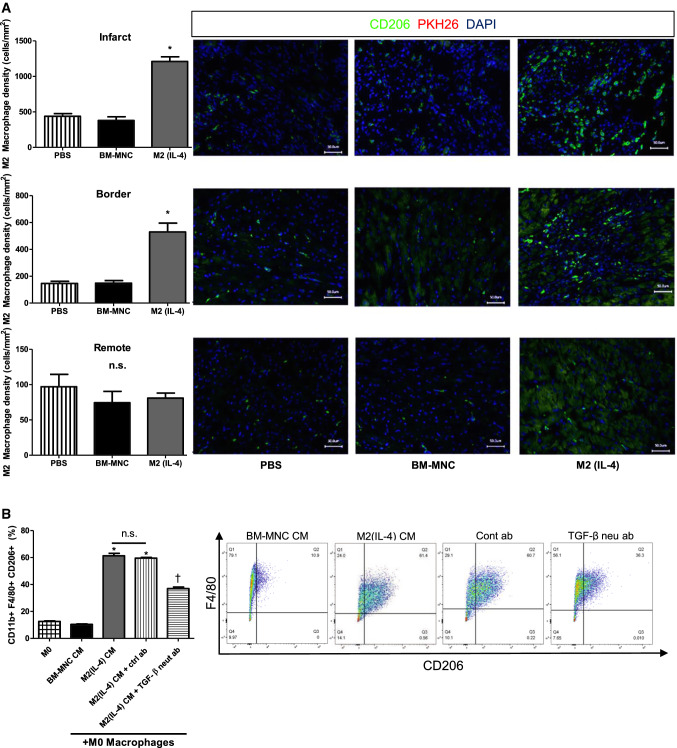
Augmented host-derived M2-like macrophages by M2(IL-4) macrophage transplantation. The potential involvement of donor M2(IL-4) macrophages in the endogenous M2-like macrophage polarization process was investigated both in vivo and in vitro. Bar charts presented as mean ± SEM. **a** Increased M2-like macrophage accumulation after M2(IL-4) macrophage transplantation. Hearts were collected at 28 days after treatment and stained for M2-like (CD206^+^) macrophages. The number of CD206^+^ macrophages was calculated in the infarct, pre-infarct border, and remote myocardium. *p < 0.05 vs. both PBS and BM-MNC groups. *N* = 4 in PBS and BM-MNC groups; *N* = 5 hearts in M2(IL-4) group. Scale bars = 50 μm. **b** Role of TGF-β1 in M2-like polarization induced by M2(IL-4) macrophages. M0 macrophages (BM-MNCs treated with M-CSF for 5 days) were subjected to different types of conditioned medium (CM) with or without neutralization antibody to TGF-β for 24 h. The phenotype changes were analyzed by flow cytometry. *ctrl ab* control antibody, *neut ab* neutralization antibody. *p < 0.05 vs. M0, BM-MNC CM and M2(IL-4) CM + TGF-β neutralization antibody groups; ^†^*p* < 0.05 vs. M0 and BM-MNC CM groups; *N* = 4 for all groups except for the M2(IL-4) CM group where *N* = 5

In addition, we found that CD206^+^ M2-like macrophages, which were PKH26^−^, thus host-derived, were accumulated primarily in the infarct area regardless of the treatment (Fig. 8a). Of note, the number of CD206^+^ cells was twice larger after M2(IL-4) macrophage transplantation in both infarct and peri-infarct border myocardium compared to both BM-MNC transplantation and control groups (Fig. 8a). There was no difference between the three groups in the remote areas of the heart, where a small number of CD206^+^ cells were present. We have previously shown that the majority of CD206^+^ cells in the heart also express CD11b and F4/80 and have a reparative phenotype [30]. Therefore, CD206^+^ cells found in the present experiment most likely were M2-like macrophages. Collectively, these results suggested that endogenous (host-derived) M2-like reparative macrophages could play a role in the paracrine effect induced by cell therapy as a secondary mediator arm [3]. Namely, transplanted M2(IL-4) macrophages would activate host-derived reparative macrophages, which in turn could provide a powerful tissue-repairing secretome possibly including cytokines, growth factors and exosomes, resulting in myocardial tissue repair.

We further investigated the mechanism of M2-like polarization of host macrophages by transplantation of M2(IL-4) macrophages. Based on the observation that *Tgfb1* was upregulated by both M2(IL-4) macrophages in vitro (Fig. 3) and post-M2(IL-4) macrophage transplantation in vivo (Fig. 7), we hypothesized that TGF-β1 may play a pivotal role in this process. To test this hypothesis, we conducted in vitro experiments using conditioned medium collected from M2(IL-4) macrophages. We found that M2(IL-4) macrophage conditioned medium was capable of polarizing M0 macrophages to an M2-like phenotype, while antibody neutralization to TGF-β1 significantly reduced this effect (Fig. 8b), suggesting a role of TGF-β1 in M2-like polarization of host macrophages by transplanted M2(IL-4) macrophages.

## Discussion

This study demonstrated that the usage of concomitant M-CSF and IL-4 treatment produced CD11b^+^F4/80^+^CD206^+^ M2-like macrophages from mouse BM-MNCs more effectively than other treatments studied. These generated M2(IL-4) macrophages were found to express and secrete a more extensive scope of reparative and anti-inflammatory factors compared to BM-MNCs. We also provided robust pre-clinical proof of concept data that transplantation of reparative macrophages, which were produced from BM-MNCs with M-CSF + IL-4 treatment, resulted in augmented therapeutic effects, compared to BM-MNC transplantation, in a murine model of MI. This was associated with enhanced tissue repair post-MI, including augmented microvascular formation, reduced cardiomyocyte hypertrophy, reduced the inflammatory gene profile of the myocardium, thicker scar area formation and reduced pathological interstitial fibrosis, in correspondence to amplified myocardial upregulation of reparative genes. Of note, survival of reparative macrophages in the heart post-transplantation was increased as compared to BM-MNCs. In addition, it was observed that transplanted M2(IL-4) macrophages augmented host-derived endogenous reparative macrophages. We, thus, speculate that this increase of endogenous reparative cells might act to further enhance cardiac repair through their own secretome. We identified that this unique macrophage-to-macrophage communication occurred through TGF-β, at least in part. These data warrant further pre-clinical and clinical development of this advanced cell therapy for MI.

After 7 days of in vitro treatment with M-CSF + IL-4, more than 90% of BM-MNCs were converted into M2-like macrophages based on CD11b, F4/80 and CD206 triple positivity. In addition, this protocol was the only one, among all tested, which achieved increased yields from the original BM-MNC number. This may be explained by the fact that IL-4 promotes macrophage proliferation in the tissues beyond the homeostatic levels controlled by M-CSF [10]. While most of the protocols in the literature use initially M-CSF as a differentiation factor from BM-MNCs to M0 (unpolarized) macrophages followed by an additional IL-4 polarization step, we have demonstrated that the use of both cytokines simultaneously for the entire duration achieved a higher purity of CD11b, F4/80 and CD206 triple positive cells after only 6 days of treatment. Freshly isolated BM-MNCs contain monocyte precursors at different maturation stages. This maturation process is tightly controlled in vivo by M-CSF [9]. It is, therefore, likely that, in vitro, the stages and pace of BM-MNC differentiation to macrophages in response to M-CSF are different. The presence of IL-4 in the culture medium right form the onset biases the differentiation towards an M2-like phenotype of newly developed M0 macrophages, thus shortening the total differentiation time.

Transplantation of M2-like macrophages generated from BM-MNCs using the M-CSF + IL-4 treatment resulted in marked improvements in cardiac function and structure post-MI, compared to BM-MNC transplantation. Our study suggested that two major factors underpinning this improved therapeutic effect were the increased quality and the enhanced quantity of donor cells. Firstly, the reparative secretome of donor cells was enhanced in M2-like macrophages. In vitro studies demonstrated that M2(IL-4) macrophages produced from BM-MNCs exhibited expression of M2-specific markers, including *Arg1*, *Fizz1* and *Ym1* and indeed showed substantially increased secretion of VEGF, IL-1ra, IGF-1 and TGF-β1. These are known to contribute to myocardial repair post-MI through neovascular formation, anti-inflammation, cardiac protection and stimulation of endogenous regeneration. In addition, *Tnfa* was downregulated in M2(IL-4) macrophages, highlighting the anti-inflammatory effect of these cells. A potential concern would be that the achieved differentiation could not be maintained longitudinally, i.e., the M2(IL-4) macrophages could drift towards a pro-inflammatory M1-like phenotype post-transplantation. However, our results confirmed that produced M2(IL-4) macrophages maintain their M2-like phenotype (in terms of expression of *Arg1*, *Fizz1*, *Ym1, Tgfb1, Igf1* and *Il1ra*) even under a hostile, inflammatory environment in vitro as well as in vivo in the post-MI heart. Transplantation of M2(IL-4) macrophages resulted in enhanced myocardial tissue repair post-MI, and this was correlated with upregulated myocardial expression of the same reparative genes as detected in vitro. Secondly, donor cell retention/survival post-transplantation was markedly improved as measured by both real-time PCR-based quantitative assessment and semi-quantitative histological investigations. While only less than 1% of the transplanted BM-MNCs were detectable, 7% of M2(IL-4) macrophages were detectable at 28 days post-transplantation. This might be explained by different cellular phenotype, including differences in size and adhesiveness. In addition, macrophages are naturally found to be localized in tissues including the myocardium whereas BM-MNCs are immature cells and may not survive well in tissues other than bone marrow. Although it is highly likely that the mechanism underpinning myocardial repair by M2(IL-4) macrophage transplantation is the paracrine effect mediated by their secretome, this study does not completely rule out involvement of cardiomyogenic differentiation or fusion of donor cells. To conclude this, further studies using specific genetic labelling and cell fate tracking are needed.

Furthermore, our results provided additional mechanistic information on the benefits of M2(IL-4) macrophage transplantation therapy. Interestingly, transplantation of donor M2(IL-4) macrophages augmented the number of endogenous, host-derived CD206^+^ M2-like macrophages in the damaged myocardium. It is likely that the host-derived cardiac M2-like macrophages added a reparative ability, as previously reported [18]. Thus, we consider that such an increased endogenous M2-like macrophage number, together with externally added M2-like macrophages, led to enhanced recovery of the damaged myocardium post-MI. Our in vitro data demonstrated that the secretome of M2(IL-4) macrophages was capable of polarizing M0 macrophages towards an M2-like phenotype and that TGF-β1 might play a role in this polarization process. This result is supported by the previous evidence that TGF-β1 can directly polarize or facilitate polarization towards an M2-like phenotype [8, 42]. However, it is likely that not only TGF-β1 but also other factors contributed to the augmentation of polarization of endogenous macrophages/monocytes towards an M2-like phenotype. In addition, this TGF-β-mediated effect is likely to be only a part of the mechanism responsible for the enhanced myocardial repair by M2(IL-4) macrophage transplantation. Further studies are needed to fully understand these complex mechanisms.

After M2(IL-4) macrophage transplantation, three types of reparative macrophages are potentially present in the heart: donor M2(IL-4) macrophage, host tissue-resident macrophages and host recruited monocyte-derived macrophages. Our immunohistolabeling study demonstrated that the vast majority of CD206^+^ cells in the myocardium were PKH26^−^ host-derived cells, and the number of PKH26^+^ donor M2(IL-4) macrophages was much smaller. Consistently, our *Sry* gene quantification showed that the presence of donor M2(IL-4) macrophages was minor (approximately 30,000 cells only in a whole heart). In addition, we and others have reported that there is drastic reduction in the number of cardiac tissue-resident macrophages after MI, and that most macrophages in the heart after day 3 post-MI are recruited from the circulation (monocyte-derived) [21, 26]. Therefore, it will be reasonable to consider that the majorly of M2-like macrophages observed after M2(IL-4) macrophage transplantation are host monocyte-derived macrophages.

This report provides pre-clinical proof of concept data that support M2(IL-4) macrophage transplantation for the treatment of MI. However, for successful clinical translation of our experimental data obtained in the animal models, a series of focused experiments need to be completed with extreme caution. These include studies accounting for differences in age, sex and co-medications, investigations using human cells and further optimisation of the cell-delivery route. It is also important to confirm the efficacy of M2(IL-4) macrophage transplantation when it is conducted at a later phase post-MI in an ischemia–reperfusion model. Our results demonstrated that the optimal duration for M2-like macrophage harvesting using our M-CSF + IL-4 pre-treatment protocol is 6 days. Although this time frame could be integrated in the current BM-MNC transplantation protocol for acute MI [22], a shorter pre-treatment period would be considered more beneficial in terms of the incurred cost and labor. Therefore, it will be useful to shorten the current 6-day length of the protocol.

In conclusion, this translational study provided robust pre-clinical evidence for transplantation of reparative macrophages for the treatment of MI, which is a refined alternative to the current BM-MNC transplantation. The M-CSF + IL-4 treatment was effective in producing reparative macrophages from BM-MNCs in vitro and this pre-treatment to donor BM-MNCs improved the therapeutic effects of cell transplantation. Further pre-clinical and clinical development of this strategy for the treatment of MI is warranted.

## Electronic supplementary material

Below is the link to the electronic supplementary material.

Supplementary material 1 (PDF 618 kb)

Supplementary material 2 (DOCX 24 kb)
