# Supplementary figures and images for: Reparative macrophage transplantation for myocardial repair: a refinement of bone marrow mononuclear cell-based therapy
Source: Basic Res Cardiol. 2019 Aug 1;114(5):34. doi: 10.1007/s00395-019-0742-1 (PMC6675756; doi:10.1007/s00395-019-0742-1)

Figure S1

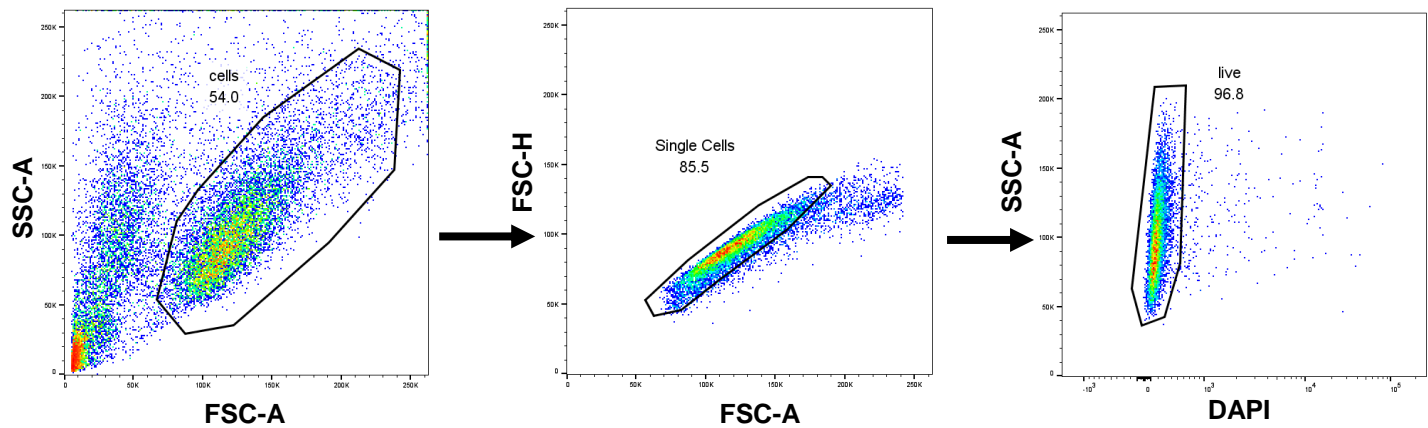

Figure S2

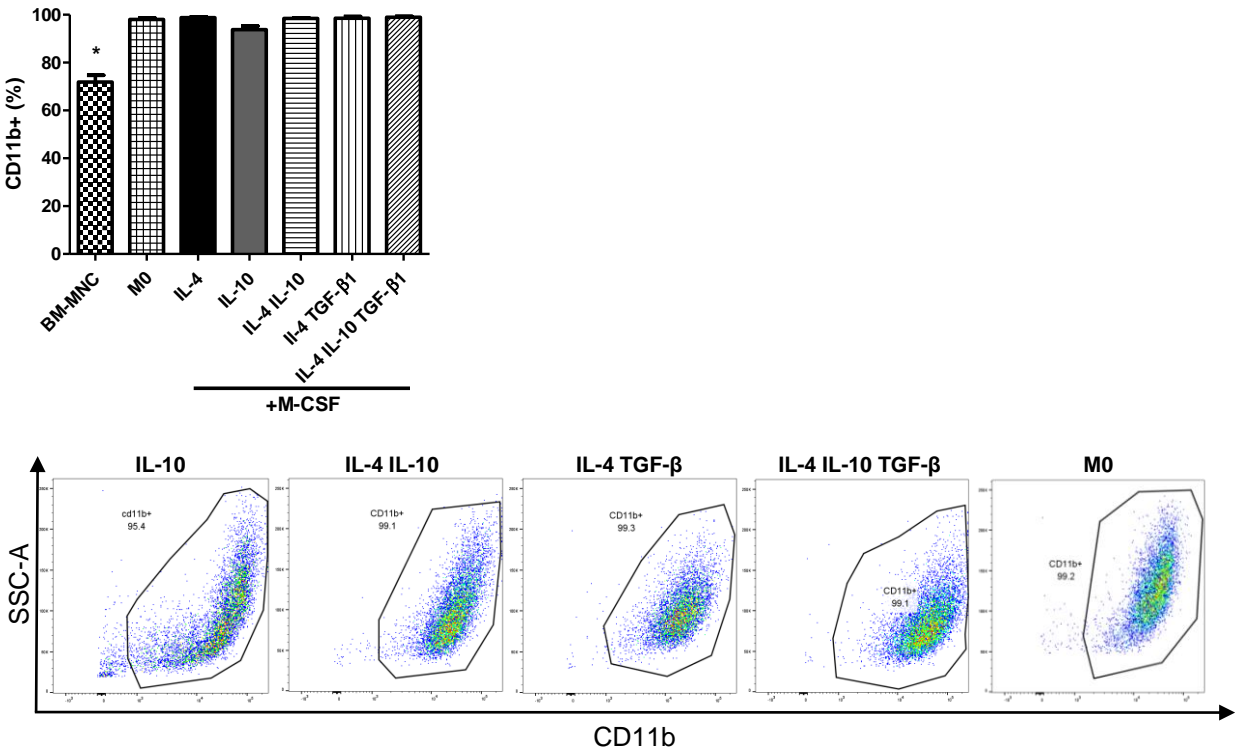

**Figure S3**

**Day 4**

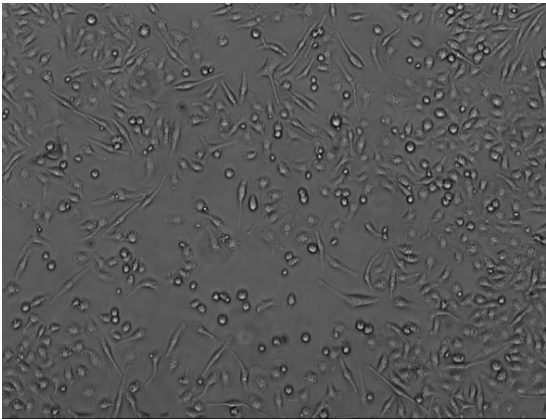

**Day 5**

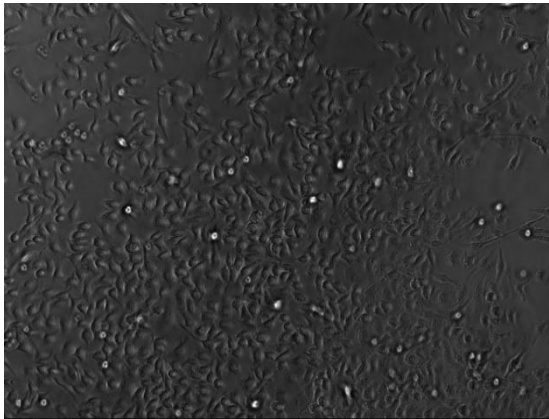

**Day 6**

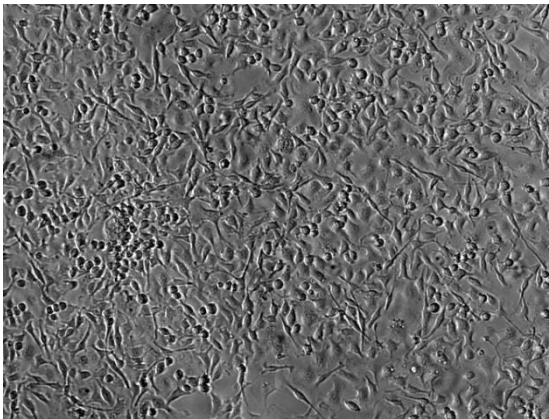

**Day 7**

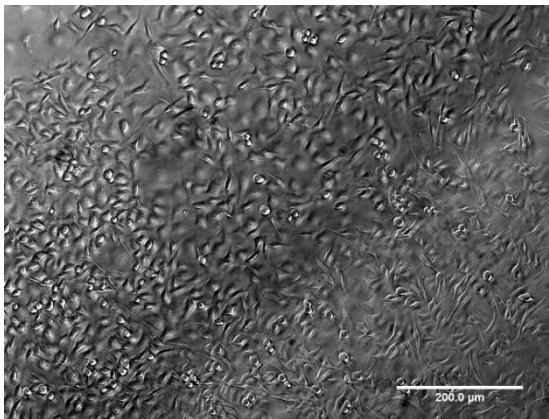

Figure S4

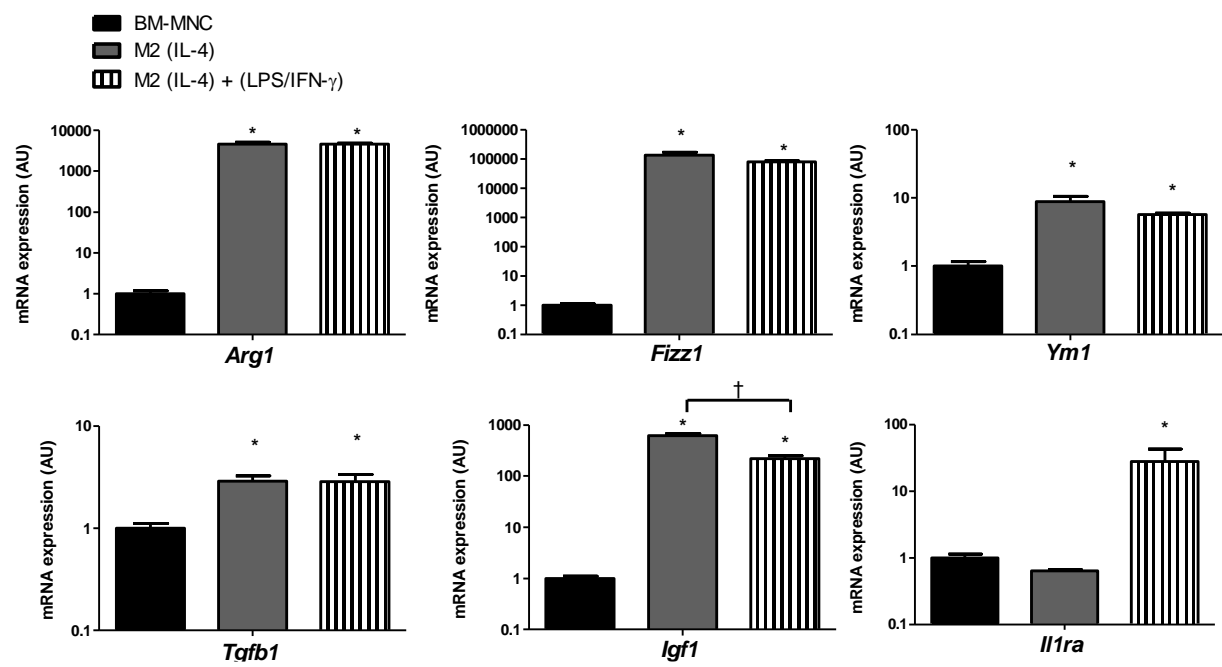

Figure S5

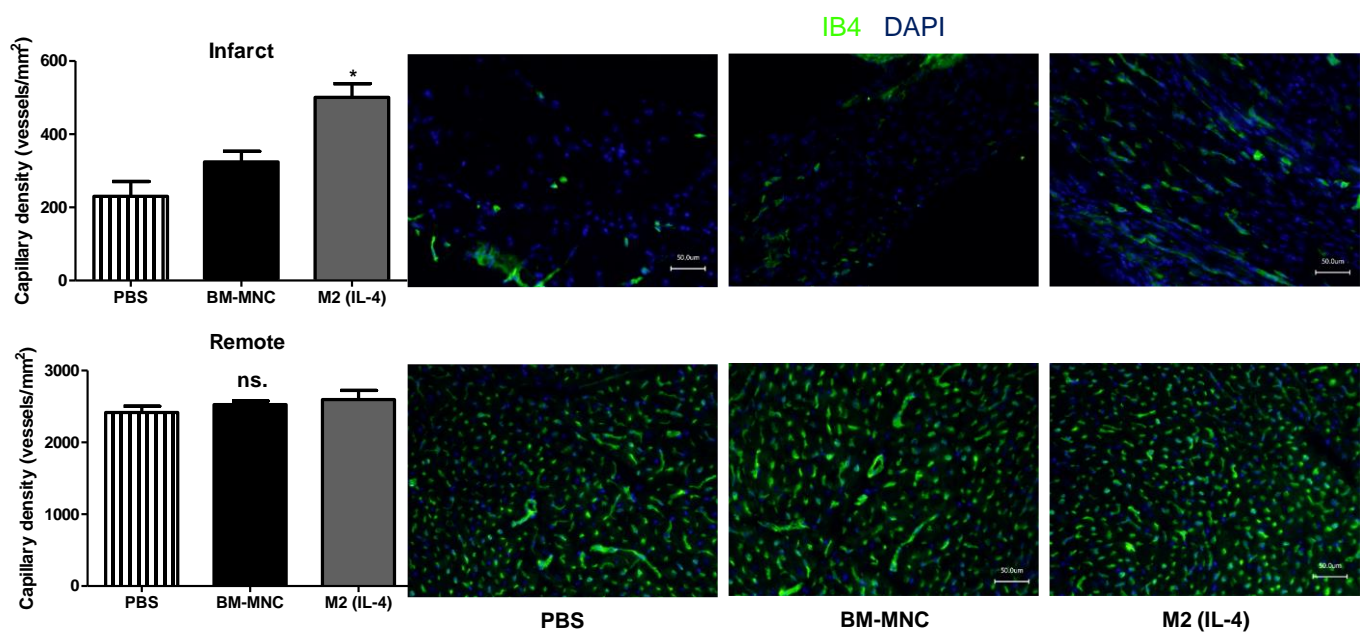

Supplement: Supplementary file 1 — Supplementary material 1 (PDF 618 kb) [file 395_2019_742_MOESM1_ESM.pdf]
