# Supplementary material for: Reparative macrophage transplantation for myocardial repair: a refinement of bone marrow mononuclear cell-based therapy
Source: Basic Res Cardiol. 2019 Aug 1;114(5):34. doi: 10.1007/s00395-019-0742-1 (PMC6675756; doi:10.1007/s00395-019-0742-1)
Supplement: Supplementary file 2 — Supplementary material 2 (DOCX 24 kb) [file 395_2019_742_MOESM2_ESM.docx]

**Supplemental Information**

**ONLINE SUPPLEMENTAL TABLES**

**Table S1. Conjugated antibodies and IgG controls used for flow cytometry**

| **Primary antibody** | **IgG Controls** |
| --- | --- |
| **APC-CD11b** (IgG2b,M1/70, monoclonal, rat) 1:1000 | **APC-IgG2b** (monoclonal, rat) 1:1000 |
| **AF488-CD206** (IgG2a,C068C2, monoclonal, rat) 1:50 | **AF488-IgG2a** (monoclonal, rat) 1:50 |
| **AF488-CD80** (IgG2a,RM80, monoclonal, rat)  1:10 | **AF488-IgG2a** (monoclonal, rat) 1:10 |
| **PE-F4/80 (**IgG2a,BM-8, monoclonal, rat**)**  1:400 | **PE-IgG2a** (monoclonal, rat)  1:400 |

**Table S2. Primer Sequence Description**

| Gene | Primer Sequence |
| --- | --- |
| *ARG1* | Forward:5'-CAAGACAGGGCTCCTTTCAG-3'  Reverse: 5'-AAGCAAGCCAAGGTTAAAGC-3' |
| *FIZZ1* | Forward:5'-AGGAACTTCTTGCCAATCCA-3'  Reverse: 5'-ACAAGCACACCCAGTAGCAG-3' |
| *YM1* | Forward:5'-TGGTGAAGGAAATGCGTAAA-3'  Reverse: 5'-GTCAATGATTCCTGCTCCCTG-3' |
| *IL-10* | Forward:5'-GGACAACATACTGCTAAAGGACTCCT-3'  Reverse: 5'-GCCTGGGGCATCACTTCTAC-3' |
| *TGFB1* | Forward:5'-CCTATATTTGGAGCCTGGACACAC-3'  Reverse: 5'-GCTTGCGACCCACGTAGTAGA-3' |
| *IGF1* | Forward:5'-GGACCGAGGGGCTTTTACTTC-3'  Reverse: 5'-GGCACAGTACATCTCCAGTCTCCTC-3' |
| *TNFA* | Forward:5'-ATGGCCTCCCTCTGATCAGTT-3'  Reverse: 5'-TCTTTGAGATCCATGCCGTTG-3' |
| *VEGF* | Forward:5'-GAGGCAGCTTGAGTGAAACGAAC-3'  Reverse: 5'-GTGACATGGTTAATCGGTCTTTCC-3' |
| *IL1RA* | Forward:5'-GTGCCTATTGACCTTCATAGTGTGTTC-3'  Reverse: 5'-GCGCTTGTCTTCTTCTTTGTTCT-3' |
| *MMP9* | Forward:5'-GCAATGTGGATGTTTTTGATGCTATT-3'  Reverse: 5'-CCTGTAATGGGCTTCCTCTATGATT-3' |
| *PTGES2* | Forward:5'-GAATCCCGTGAGAAGGACTGAGA-3'  Reverse: 5'-CTTGAGGGCACTAATGATGACAGAG -3' |
| *MMP2* | Forward:5'-CTTGACCAGAACACCATCGAGAC-3'  Reverse: 5'-GTGTGTAACCAATGATCCTGTATGTGA-3' |
| *TIMP1* | Forward:5'-TATAGTGCTGGCTGTGGGGTGT-3'  Reverse: 5'-AAGCAAAGTGACGGCTCTGG-3' |
| *SPP1* | Forward:5'-GATAGCTTGGCTTATGGACTGAGG-3'  Reverse: 5'-GACTCCTTAGACTCACCGCTCTT -3' |

**Table S3. Primary and secondary antibody selection for immunofluorescent staining**

| **Primary antibody** | **Secondary antibody** |
| --- | --- |
| **Isolectin B4 (Biotinilated)**  (GSL I-B4) 1:100 | **Streptavidin-AF488**  1:300 |
| **α-sarcomeric actinin**  (IgG1,EA-53,monoclonal,mouse)  1:100 | **AF647 donkey anti-mouse (IgG H+L)** |
| **Wheat Germ Agglutinin - FITC (WGA)**  1:100 | **N/A** |
| **CD206** (IgG,C-20, polyclonal, goat) 1:100 | **AF488 donkey anti-goat (IgG H+L)** 1:300 |

**ONLINE SUPPLEMENTAL FIGURE LEGENDS**

***Figure S1. Gating strategy for flow cytometry procedures.*** Routine gating strategy utilised in all experiments for exclusion of debris, doublets and dead cells. Representative images from an M2(IL-4) macrophage sample at day 7.

***Figure S2.*** ***CD11b^+^ lineage purity after 7 days of culture with each polarization protocol.*** Quantitative and representative figures depicting the CD11b^+^ purity of BM-MNCs treated with various combinations of cytokines. Bar charts presented as mean ± SEM. **= significant vs. all other groups; p<0.05. N=4 for IL-10, IL-4/IL-10, IL-4/TGF-β, IL-4/IL-10/ TGF-β and M0; N=8 for IL-4 and N=6 for BM-MNC*.

***Figure S3. Representative M2(IL-4) macrophage images during the polarization protocol.*** Representative images from day 4, 5, 6 and 7 of the *in vitro* M-CSF + IL-4 protocol. Scale bar= 200μm.

***Figure S4. M2-like macrophage specific gene profile is maintained after inflammatory stimulation.*** M2(IL-4) macrophages were stimulated with LPS and IFN-γ for 6 hours. Gene expression by real time qPCR was assessed for three key M2-like markers (*Arg1, Fizz1, Ym1*) and three functional genes (*Tgfb1, Igf1, Il1ra*)*.* Bar charts presented as mean ± SEM*.*= significant vs. BM-MNC; †= significant vs. M2(IL-4)+ LPS/IFN-γ; p<0.05.* Data pooled from 6-12 independent biological samples with 3 technical replicates.

***Figure S5. Increased capillary density in the infarct area after M2(IL-4) macrophage transplantation.*** There was no change in capillary numbers in the remote area. Bar charts presented as mean ± SEM. **= significant vs. both PBS and BM-MNC; p<0.05****.*** Data pooled from 4 (PBS and BM-MNC) or 5 hearts in M2(IL-4) group.
